# Reciprocal regulation between ER stress and autophagy in renal tubular fibrosis and apoptosis

**DOI:** 10.1038/s41419-021-04274-7

**Published:** 2021-10-29

**Authors:** Shaoqun Shu, Hui Wang, Jiefu Zhu, Zhiwen Liu, Danyi Yang, Wenwen Wu, Juan Cai, Anqun Chen, Chengyuan Tang, Zheng Dong

**Affiliations:** 1 Department of Nephrology, Hunan Key Laboratory of Kidney Disease and Blood Purification, The Second Xiangya Hospital at Central South University, Changsha, 410011 China; 2 Department of Cellular Biology and Anatomy, Medical College of Georgia at Augusta University and Charlie Norwood VA Medical Center, Augusta, GA USA

**Keywords:** Acute kidney injury, Chronic kidney disease

## Abstract

Both endoplasmic reticulum (ER) stress and autophagy have been implicated in chronic kidney injury and renal fibrosis. However, the relationship and regulatory mechanisms between ER stress and autophagy under this condition remain largely unknown. In this study, we first established a mouse model of ER stress-induced chronic kidney injury by 2 weekly injections of a low dose of tunicamycin (TM), a classical ER stress inducer. This model showed the induction of ER stress, autophagy, fibrosis and apoptosis in kidney tissues. In vitro, TM also induced ER stress, autophagy, fibrosis and apoptosis in HK-2 human kidney proximal tubular cells and BUMPT-306 mouse kidney proximal tubular cells. In these cells, autophagy inhibitor suppressed TM-induced fibrotic changes and apoptosis, suggesting an involvement of autophagy in ER stress-associated chronic kidney injury. PERK inhibitor ameliorated autophagy, fibrotic protein expression and apoptosis in TM-treated cells, indicating a role of the PERK/eIF2α pathway in autophagy activation during ER stress. Similar results were shown in TGF-β1-treated HK-2 cells. Interestingly, in both TM- or TGF-β1-treated kidney proximal tubular cells, inhibition of autophagy exaggerated ER stress, suggesting that autophagy induced by ER stress provides a negative feedback mechanism to reduce the stress. Together, these results unveil a reciprocal regulation between ER stress and autophagy in chronic kidney injury and fibrosis.

## Introduction

Endoplasmic reticulum (ER) stress is a cellular state with ER dilatation due to the accumulation of misfolded or unfolded proteins, which results in unfolded protein response (UPR) [1–3]. In resting cells, the ER stress sensors (ATF6, IRE1, and PERK) are inactive due to their sequestration by the ER chaperone binding immunoglobulin protein (BiP). Upon ER stress, BiP dissociates from ER stress sensors and binds to misfolded or unfolded proteins, leading to the activation of three major signaling pathways of UPR that are characterized respectively by the activation of ATF6, IRE1–XBP1, and PERK–eIF2α [1–3].

Autophagy is a cellular process of “self-eating” that degrades the bulk of cytoplasmic components, including misfolded protein aggregates, damaged organelles, and foreign organisms, through the formation of autophagosomes and autolysosomes [4, 5]. A link or association between ER stress and autophagy has been suggested. For example, ER constitutes the principal source of autophagic isolation membrane [6], while damaged ER may be removed via the selective form of autophagy named “ER-phagy” [7]. In addition, ER stress or UPR can cooperate with autophagy to remove unfolded or misfolded proteins [8]. However, the relationship and reciprocal regulatory mechanisms between ER stress and autophagy remain largely unclear.

Renal tubulointerstitial fibrosis (TIF) is a common pathological feature in virtually all progressive and chronic kidney diseases (CKD), which affect half of the adults over age 70 and 10% of the general population worldwide. Appearing as scars, TIF is characterized by the deposition of fibrotic matrix and expansion of myofibroblasts in kidney peritubular interstitium. The development of TIF is a complex process involving various intra- and extra-renal cell types, and changes of the micro-environment in kidney tissues [9–13]. It is generally understood that tubular atrophy and degeneration result in the release of pro-inflammatory and profibrotic factors for the initiation and progression of TIF [9–13]. Nonetheless, the mechanism underlying the pro-fibrotic phenotype in renal tubular cells is multi-faceted and remains unclear.

ER stress and autophagy have been implicated in the development of renal fibrosis [5, 14–18]. However, the role of ER stress in renal fibrosis and its underlying mechanism remains largely unclear. Moreover, the relationship between ER stress and autophagy, and their reciprocal regulation in renal fibrosis remain to be investigated. In this study, by using a mouse model treated with the classical ER stress inducer tunicamycin (TM) and in vitro proximal tubular cells treated by TM or transforming growth factor-β1 (TGF-β1), we have provided evidence that ER stress may directly induce renal fibrosis. We further demonstrate that the PERK-mediated UPR signaling pathway links ER stress to the autophagy activation, autophagy activation contributes at least partially to ER stress-associated fibrosis, and ER stress-activated autophagy may represent a negative feedback mechanism to reduce the stress. Collectively, this study unveils a reciprocal regulation between ER stress and autophagy in chronic kidney injury and fibrosis.

## Results

### Tunicamycin (TM) induces ER stress, autophagy, fibrosis, and apoptosis in kidneys of C57BL/6 mice

**Fig. 1:**
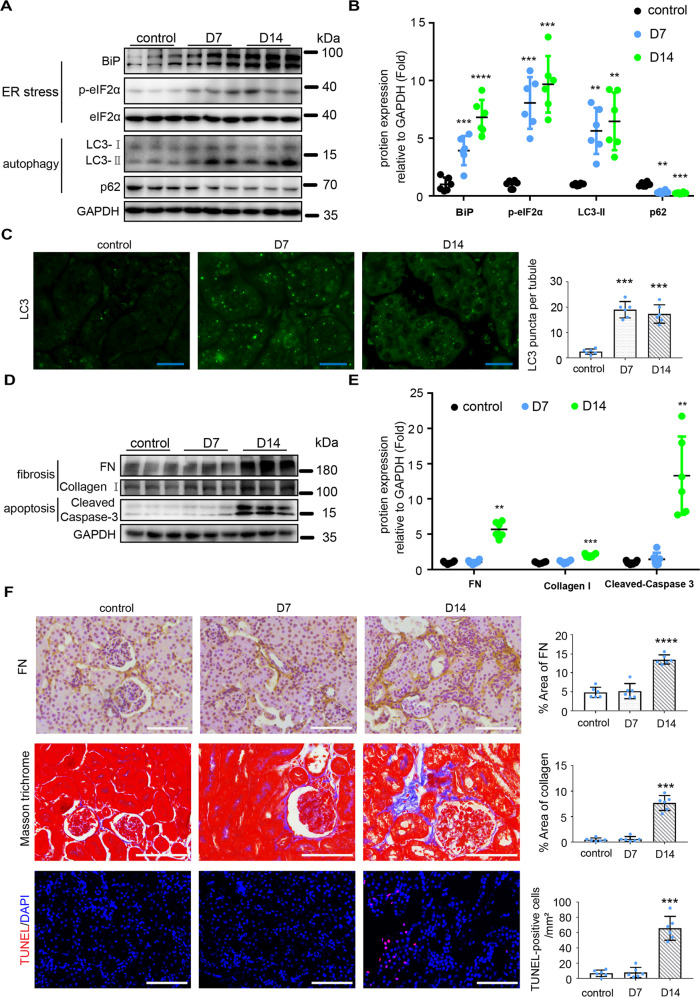
TM induces ER stress, autophagy, fibrosis and apoptosis in mouse kidneys. C57BL/6 mice (male, 8–10 weeks) were subjected to two weekly injections of 0.25 mg/kg TM (or DMSO for control) to collect kidney tissues 7 days or 14 days later. **A**, **B** Immunoblot analysis of proteins in kidney cortical tissues to indicate ER stress and autophagy. For quantification, the proteins bands were analyzed by densitometry and their signals were normalized with that of GAPDH and values expressed relative to mean of control counterparts. **C** Representative images and quantification of the staining of LC3. Bar = 20 μm. **D**, **E** Immunoblot analysis of fibrosis marker proteins and cleaved caspase-3 in kidney cortical tissues. For quantification, the proteins bands were analyzed by densitometry and their signals were normalized with that of GAPDH and values expressed relative to mean of control counterparts. **F** Representative images and quantification of the staining of fibronectin (FN), Masson trichrome, and TUNEL assay. Bar = 100 μm. Quantitative data are expressed as mean ± SD. *n* = 6. **p < 0.01; ***p < 0.001; ****p < 0.0001 vs. control.

ER stress and autophagy have been recently implicated in renal fibrosis and apoptosis [5, 14–18]. However, the interaction between ER stress and autophagy, and the underlying mechanism are largely unknown. To explore whether ER stress can induce renal autophagy, fibrosis, and apoptosis in mice, we tested the effect of tunicamycin (TM), a classical ER stress inducer, in C57BL/6 mice. TM activates ER stress through specifically inhibiting N-glycosylation [19]. Single intraperitoneal injection of 1 or 2 mg/kg of TM led to acute kidney injury in C57BL/6 mice [20–22], but the chronic effect of TM in kidneys, such as renal fibrosis, has not been reported. We first tested the effect of 1, 2, or 4 mg/kg TM in mice, and found that these dosages of TM led to significant animal death within 21 days of observation (Fig. S1), indicating lethal toxicity of TM at ≥1 mg/kg concentrations in mice. We then tested the effects of two weekly injections of 0.25 or 0.5 mg/kg TM. Because 0.5 mg/kg TM led to a marked weight loss (data not shown), we utilized the strategy of 2 weekly injections of 0.25 mg/kg of TM in followed studies. As shown in Fig. 1A, B, 0.25 mg/kg of TM significantly increased the levels of BiP and phosphorylated eIF2α (p-eIF2α), indicating induction of ER stress. Autophagy was also activated at both day 7 and day 14 post-TM administration as manifested by the accumulation of autophagosome marker protein LC3-II, and decrease of the autophagy substrate protein p62 (Fig. 1A, B). Consistently, increases of granular staining of LC3 indicative of autophagosomes were detected in the renal proximal tubular cells of TM-treated mice compared with control mice (Fig. 1C and Fig. S2). Interestingly, renal interstitial fibrosis appeared only at day 14 after TM treatment as indicated by the increased expression of fibrotic marker proteins including Fibronectin (FN) and Collagen I, and the increased signal intensity of Masson trichrome staining (Fig. 1D–F). Renal tubular cell apoptosis was also only detected at day 14 after TM treatments as indicated by the significant increases of cleaved Caspase-3 and Tdt-mediated dUTP nick-end labeling assay (TUNEL)-positive cells (Fig. 1D–F). Together, these results suggest that repeated treatment with a low dose of TM induced ER stress, autophagy, renal fibrosis, and apoptosis.

### TM sequentially induces ER stress, autophagy, fibrosis and apoptosis in HK-2 cells

We also performed in vitro experiments to determine the effect of TM on ER stress, autophagy, fibrosis, and apoptosis in HK-2 human kidney proximal tubular cells. We initially evaluated the effect of different concentrations of TM (50, 100, 200, or 300 nM) on cell morphology. As shown in Fig. S3A, the cells exposed to 50, 100, or 200 nM TM for 24 h showed a spindle-shaped, fibroblast-like morphology in comparison to the “cobblestone” morphology of control cells. 300 nM TM caused a dramatic cell loss. TM exposure increased the levels of BiP and LC3-II, reduced the levels of p62 (Fig. S3B, C), and increased the levels of FN and Collagen I as well as the signal intensity of Collagen I staining (Fig. S3B–E). Notably, 100 nM TM caused a maximal pro-fibrotic effect. TM also induced apoptosis in HK-2 cells in a dose-dependent manner as evidenced by the increased levels of cleaved Caspase-3 (Fig. S3B and C).

**Fig. 2:**
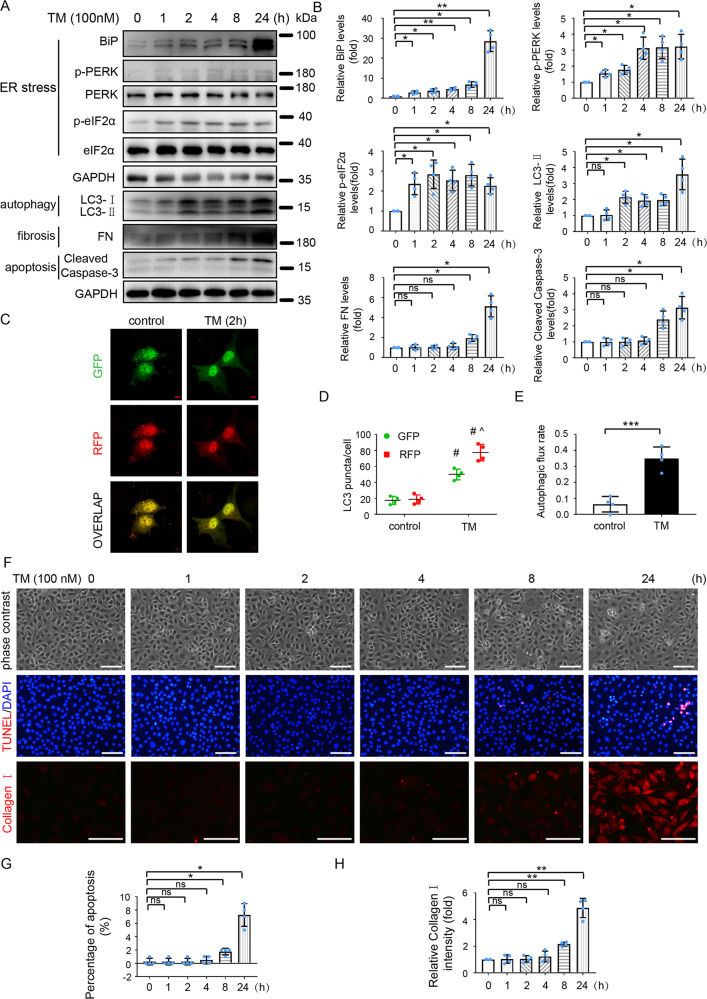
TM sequentially induces ER stress, autophagy, fibrosis, and apoptosis in HK-2 cells. **A**, **B** HK-2 cells were exposed to TM (100 nM) for various time (0, 1, 2, 4, 8, and 24 h). DMSO was used as vehicle control. Cell lysates were analyzed for BiP, p-PERK, PERK, p-eIF2α, eIF2α, LC3, FN, Cleaved Caspase-3, and GAPDH by immunoblot analysis (**A**) and quantified by densitometry (**B**). **C–E** HK-2 cells were transfected with RFP-GFP-LC3 and then treated with 100 nM TM or DMSO (vehicle control) for 2 h. **C** Representative fluorescence microscopy images showing GFP and RFP LC3 puncta in HK-2 cells. Bar = 5 μm. **D** Quantitative analysis of GFP and RFP LC3 puncta. Data are expressed as mean ± SD. ^#^*P* < 0.05 vs. control; ^*P* < 0.05 vs GFP-LC3 puncta in TM-treated group. **E** Analysis of autophagic flux rate. **F–H** Cells were incubated with 100 nM TM for 0–24 h for phase contrast and TUNEL staining and immunofluorescence analysis of Collagen I. (**F**) Representative images. Scale bar = 100 μm. **G**, **H** Quantification of TUNEL staining positive cells and Collagen I intensity. Data are expressed as mean ± SD. *n* = 4. *p < 0.05, **p < 0.01, ***p < 0.001, ns, not significant. vs. control.

To assess the temporal relationships among ER stress, autophagy, fibrosis, and apoptosis, we analyzed the time courses of these changes in HK-2 cells after TM exposure. As shown in Fig. 2A, B, 100 nM TM increased the levels of BiP, p-PERK, and p-eIF2α starting from 1 h, indicating a rapid ER stress induction by TM. Autophagy was activated at 2 h after TM treatment, as indicated by the increase of LC3-II (Fig. 2A, B). To further verify autophagy activation during TM treatment, we transiently expressed mRFP (red fluorescent protein)-GFP (green fluorescent protein)-LC3 in cells to evaluate autophagy flux. The puncta with RFP- and GFP-overlapping fluorescence signals in cell cytoplasm represent autophagosomes, whereas those with only RFP fluorescence are autolysosomes. Autophagic flux is quantitated by dividing the number of autolysosomes with the total number of both autophagosomes and autolysosomes [16, 23]. As shown in (Fig. 2C–E), the number of autophagosomes and autolysosomes as well as autophagic flux rate were significantly increased by 2 h of TM treatment, further indicating the rapid activation of autophagy. Of note, there was no obvious fibrotic changes or apoptosis until 8 h of TM treatment, as determined by phase-contrast images, measuring the levels of fibrosis marker proteins (FN and Collagen I) and apoptosis marker (Cleaved Caspase-3), and TUNEL staining (Fig. 2A, B, F-H).

Taking together, these results indicate that low concentrations of TM trigger a sequential induction of ER stress, autophagy, fibrosis, and apoptosis in HK-2 cells.

### Inhibition of autophagy attenuates TM-induced fibrosis and apoptosis in HK-2 and BUMPT-306 cells

**Fig. 3:**
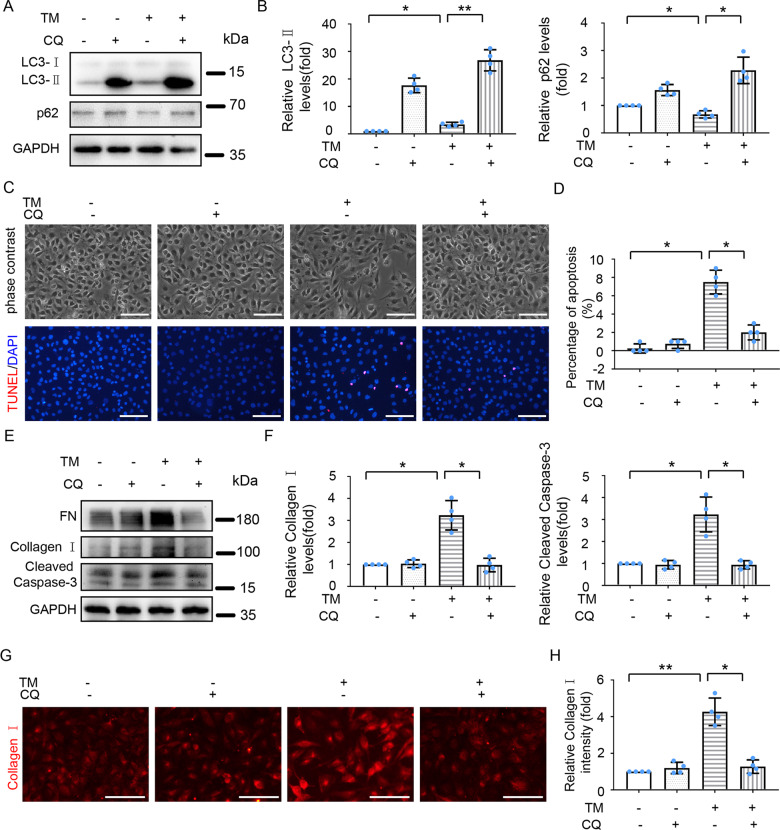
Inhibition of autophagy attenuates TM -induced fibrosis and apoptosis in HK-2 cells. HK-2 cells were treated with or without TM (100 nM) in the absence or presence of chloroquine (CQ, 20 μM) for 24 h. **A**, **B** Cell lysates were analyzed for LC3 and p62 by immunoblot analysis and quantified by densitometry. **C** Representative phase-contrast images and TUNEL staining of HK-2 cells. Scale bar = 100 μm. **D** Counting of TUNEL positive cells. **E–F** Cell lysates were analyzed for levels of FN, Collagen I, and Cleaved Caspase-3 by immunoblot analysis and quantified by densitometry. **G** Representative images of Collagen I immunofluorescence staining. Scale bar = 100 μm. **H** Quantitative analysis of Collagen I immunofluorescence. Quantitative data are expressed as mean ± SD. *n* = 4. *p < 0.05, **p < 0.01.

Since TM-induced sequential activation of ER stress, autophagy, fibrosis, and apoptosis was observed, we hypothesized that ER stress might induce fibrosis and apoptosis by activating autophagy. To test this hypothesis, we assessed the effect of chloroquine (CQ), a commonly used autophagy inhibitor that prevents autolysosomal degradation [23]. CQ treatment significantly increased LC3-II and prevented p62 degradation in both control and TM-treated HK-2 cells, verifying the autophagy inhibitory effect of CQ (Fig. 3A and B). Importantly, CQ partially suppressed TM-induced change to spindle-shaped, fibroblast-like morphology, and reduced the number of TUNEL-staining positive cells during TM treatment (Fig. 3C, D). Consistently, CQ attenuated TM-induced expression of FN, Collagen I, and cleaved Caspase-3 (Fig. 3E–H). In consistent with the findings in HK-2 cells, inhibition of autophagy by CQ partially suppressed TM-induced morphological changes and expression of FN and cleaved Caspase-3 in BUMPT-306 mouse kidney proximal tubular cells (Fig. S4). Collectively, these findings support the role of autophagy in ER stress-associated fibrosis and apoptosis following TM treatment.

### Inhibition of PERK ameliorates TM-induced autophagy, fibrosis, and apoptosis in HK-2 and BUMPT-306 cells

**Fig. 4:**
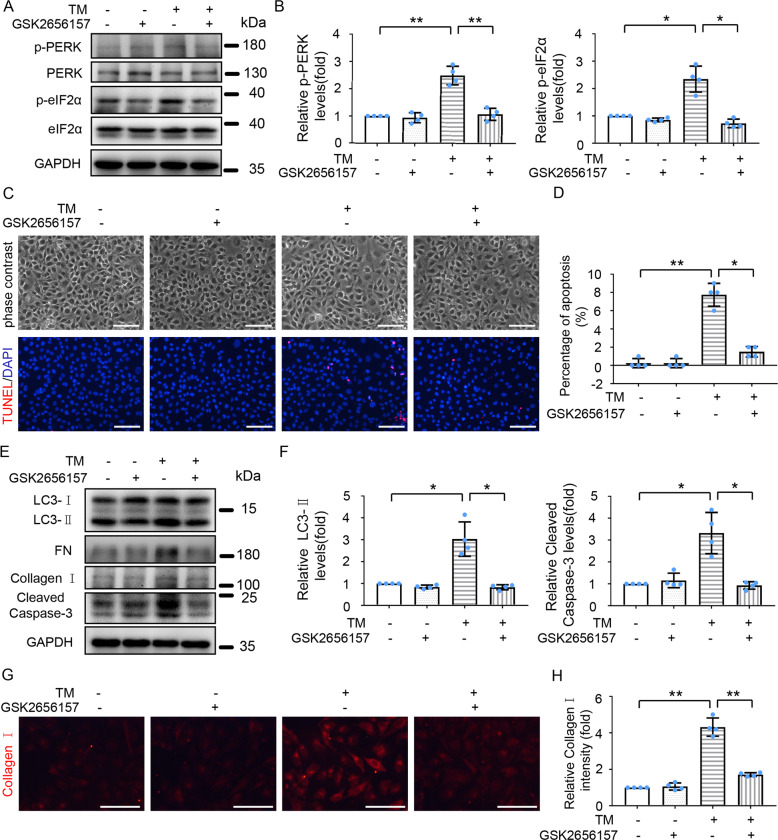
Inhibition of PERK mitigates autophagy, fibrosis, and apoptosis during TM treatment of HK-2 cells. HK-2 cells were treated with or without TM (100 nM) in the absence or presence of 1 μM GSK2656157 for 24 h. **A** Cell lysates were analyzed for p-PERK, PERK, p-eIF2α, eIF2α, and GAPDH by Immunoblot analysis; **B** Densitometric analysis of p-PERK and p-eIF2α band signals. **C** Representative phase-contrast and TUNEL staining images of HK-2 cells. Scale bar = 100 μm. **D** Counting of TUNEL staining positive cells. **E** Cell lysates were analyzed for LC3-II, FN, Collagen I, cleaved Caspase-3, and GAPDH by Immunoblot. **F** Densitometric analysis of LC3-II and cleaved Caspase-3 intensity. **G** Representative images of Collagen I immunofluorescence. Scale Bar = 100 μm. **H** Quantitative analysis of Collagen I immunofluorescence. Data are expressed as mean ± SD. *n* = 4. *p < 0.05, **p < 0.01.

The PERK**-**eIF2α pathway of UPR was activated in renal tubular cells upon TM treatment both in vivo (Fig. 1A, B) and in vitro (Fig. 2A, B), we thus wondered whether activation of this pathway contributes critically to autophagy induction, fibrosis, and apoptosis during TM treatment. To this end, we pretreated HK-2 cells with the PERK-specific inhibitor GSK2656157, which effectively inhibits PERK autophosphorylation by interacting with the PERK kinase domain [24]. GSK2656157 treatment suppressed TM -induced phosphorylation of PERK and eIF2α (Fig. 4A and B), and ameliorated TM-induced morphological changes (Fig. 4C). GSK2656157 also attenuated TM-induced expression of LC3-II (Fig. 4E, F), FN, and Collagen I (Fig. 4E, G, H), and dramatically reduced the number of TUNEL-positive cells and the levels of cleaved Caspase-3 in cells exposed to TM (Fig. 4C–F). In line with the findings in HK-2 cells, inhibition of PERK activity by GSK2656157 partially suppressed TM-induced morphological changes and ameliorated the expression of LC3-II, FN, and cleaved Caspase-3 in BUMPT-306 cells (Fig. S5). Taken together, these findings suggest that TM-induced ER stress promotes autophagy and subsequent fibrosis and apoptosis mainly through the PERK-eIF2α pathway.

### TGF-β1 induces sequential activation of ER stress, autophagy, fibrosis and apoptosis in HK-2 cells

We further investigated the relationship of ER stress with autophagy, fibrosis, and apoptosis in HK-2 cells exposed to TGF-β1, a critical factor in renal fibrosis [25]. As illustrated in Fig. S6, TGF-β1 dose-dependently induced ER stress, autophagy, fibrosis, and apoptosis in HK-2 cells within 24 h. We further examined the time courses of these TGF-β1-induced changes. ER stress was induced from 1 h after TGF-β1 treatment as indicated by the induction of BiP, and autophagy was activated from 2 h after treatment as suggested by the increases of LC3-II levels (Fig. S7A, B) and autophagic flux (Fig. S7C–E). Fibrotic marker proteins (FN and Collagen I) were induced from 4 h of TGF-β1 treatment (Fig. S7A, B, F–G), while apoptosis was first detected at 8 h after treatment as evidenced by the increases of cleaved caspase-3 levels and TUNEL-positive cells (Fig. S7A, B, F, and H). Together, these results suggest that TGF-β1 may trigger a sequential activation of ER stress, autophagy, fibrosis, and apoptosis in HK-2 cells.

### Inhibition of ER stress alleviates TGF-β1-induced autophagy, fibrosis, and apoptosis in HK-2 cells

**Fig. 5:**
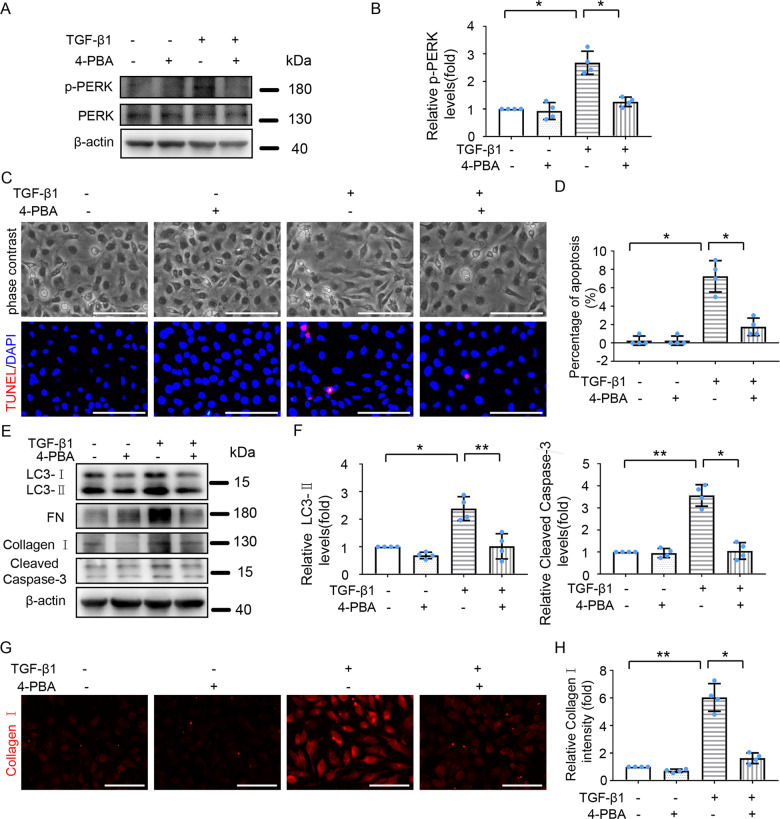
Inhibition of ER stress attenuates TGF-β1-induced autophagy, fibrosis and apoptosis in HK-2 cells. HK-2 cells were treated with or without TGF-β1 (5 ng/ml) for 24 h in the absence or presence of 4-phenyl butyric acid (4-PBA,1 mM). **A** Representative immunoblot analysis of p-PERK, PERK, and β-actin. **B** Densitometric analysis of p-PERK band intensity. **C** Representative phase-contrast and TUNEL staining images. Scale bar = 100 μm. **D** Counting of TUNEL staining positive cells. **E** Cell lysates were analyzed for LC3, FN, Collagen I, and cleaved Caspase-3 by immunoblot. **F** Densitometric analysis of LC3-II and cleaved caspase-3. **G** Representative images of Collagen I immunofluorescence staining. Scale bar = 100 μm. **H** Quantitative analysis of Collagen I immunofluorescence intensity. Data are expressed as mean ± SD. *n* = 4. *p < 0.05, **p < 0.01.

To determine the role of ER stress in TGF-β1-induced autophagy, fibrosis, and apoptosis in HK-2 cells, we tested the effect of 4-phenyl butyric acid (4-PBA), a molecular chaperon that inhibits ER stress by enhancing protein folding [7]. As shown in Fig. 5A, B, 4-PBA decreased TGF-β1-induced PERK phosphorylation. TGF-β1 treatment promoted HK-2 cells from a cobblestone morphology to a spindle-shaped, fibroblast-like state, and the TGF-β1-induced morphological change was partially abrogated by 4-PBA (Fig. 5C). 4-PBA also reduced TGF-β1-induced expression of FN and Collagen I (Fig. 5E, G, H). These findings suggest that ER stress contributes to TGF-β1-induced fibrotic changes. 4-PBA suppressed TGF-β1-induced expression of LC3-II levels (Fig. 5E, F), indicating that ER stress contributes to autophagy activation during TGF-β1 treatment. Moreover, the results of TUNEL staining and immunoblot of cleaved Caspase-3 (Fig. 5C–F) revealed that 4-PBA reduced TGF-β1-induced apoptosis. Collectively, these results support the role of ER stress in TGF-β1-induced autophagy, and subsequent renal fibrotic changes and apoptosis in HK-2 cells.

### Inhibition of autophagy attenuates fibrotic changes and apoptosis induced by TGF-β1 in HK-2 cells

**Fig. 6:**
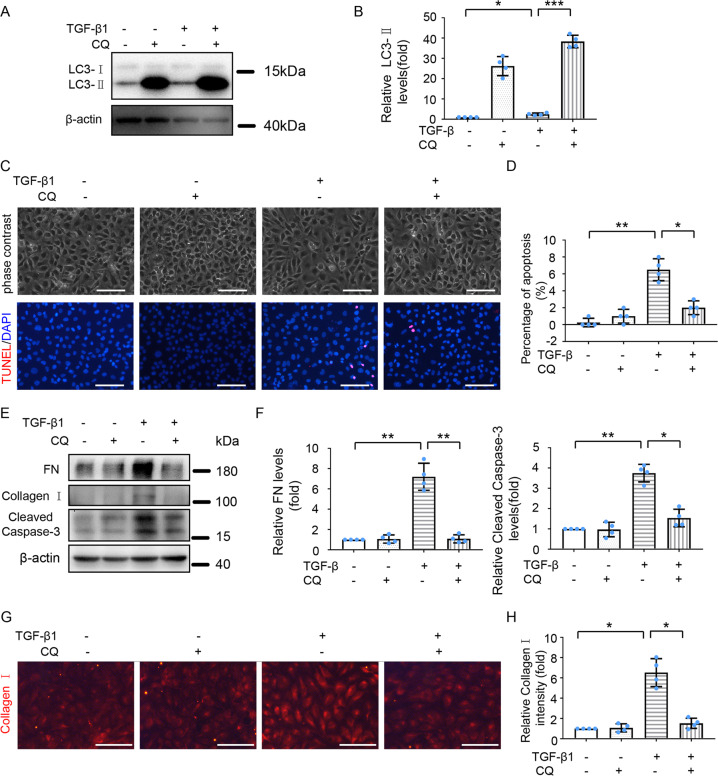
Inhibition of autophagy ameliorates TGF-β1 induced kidney fibrosis and apoptosis in HK-2 cells. HK-2 cells were treated with or without TGF-β1 (5 ng/ml) for 24 h in the absence or presence of CQ (20 μM) for 24 h. **A** Cell lysates were analyzed for LC3 and β-actin by immunoblot analysis. **B** Densitometric analysis of LC3-II band signal. **C** Representative phase-contrast and TUNEL staining images. Scale bar = 100 μm. **D** Counting of TUNEL staining positive cells. **E** Cell lysates were analyzed for FN, Collagen I, and cleaved Caspase-3 by Immunoblot analysis. **F** Densitometric analysis of FN and cleaved Caspase-3 immunoblots relative to β-actin. **G** Representative images of Collagen I immunofluorescence staining. Scale bar = 100 μm. **H** Quantitative analysis of Collagen I immunofluorescence intensity. Data are expressed as mean ± SD. *n* = 4. *p < 0.05; **p < 0.01; ***p < 0.001.

To verify whether ER stress induces fibrosis and apoptosis through activating autophagy, we assessed the effect of autophagy inhibition on fibrosis and apoptosis during TGF-β1 treatment. To this end, HK-2 cells were pre-treated with CQ for 1 h, followed by TGF-β1 (5 ng/ml) treatment for 24 h. As shown in Fig. 6A–B, CQ tremendously enhanced TGF-β1-induced LC3-II accumulation, verifying the inhibitory effect of CQ on autophagy. CQ obviously ameliorated TGF-β1-induced morphological changes of HK-2 cells (Fig. 6C), and consistently, CQ markedly decreased the expression of fibrotic marker proteins like FN and Collagen I (Fig. 6E–H). Meanwhile, CQ attenuated TGF-β1-induced increases of TUNEL positive cells (Figs. 6C, D) and Cleaved Caspase-3 levels (Fig. 6E, F). Thus, inhibition of autophagy attenuated TGF-β1 induced fibrosis and apoptosis in HK-2 cells.

### Inhibition of PERK ameliorates TGF-β1-induced autophagy, fibrotic changes, and apoptosis in HK-2 cells

**Fig. 7:**
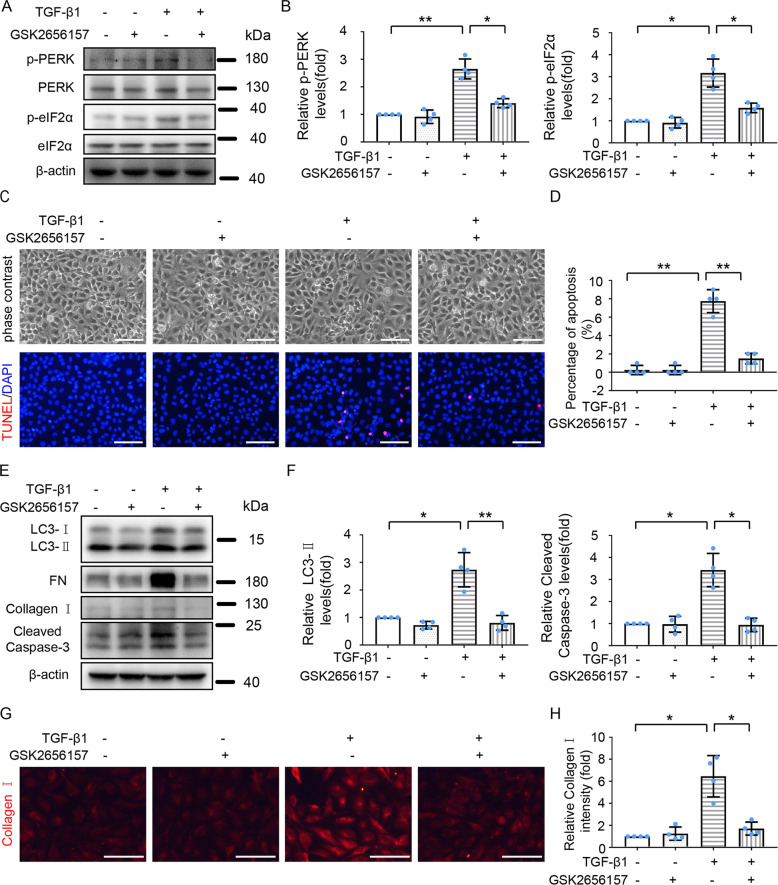
PERK inhibitor suppresses TGF-β1-induced autophagy, fibrosis, and apoptosis in HK-2 cells. HK-2 cells were treated with or without TGF-β1 (5 ng/ml) for 24 h in the absence or presence of 1 μM GSK2656157. **A** Cell lysates were analyzed for levels of p-PERK, PERK, p-eIF2α, eIF2α, and β-actin by immunoblot analysis. **B** Densitometric analysis of p-PERK and p-eIF2α immunoblots relative to β-actin. **C** Representative phase-contrast and TUNEL staining images. Scale bar = 100 μm. **D** Counting of TUNEL-staining positive cells. **E** Cell lysates were analyzed for LC3, FN, Collagen I, cleaved Caspase-3, and β-actin by Immunoblot analysis. **F** Densitometric analysis of LC3-II and cleaved Caspase-3 blots relative to β-actin. **G** Representative images of Collagen I immunofluorescence staining. Scale bar = 100 μm. **H** Quantitative analysis of Collagen I immunofluorescence intensity. Data are expressed as mean ± SD. *n* = 4. *p < 0.05; **p < 0.01.

Activation of the PERK**-**eIF2α signaling pathway contributes to TM-induced autophagy, fibrosis, and apoptosis (Fig. 4). We thus determined whether this pathway was also involved in TGF-β1-induced autophagy and subsequent fibrosis and apoptosis via inhibiting PERK activity with GSK2656157. As expected, GSK2656157 suppressed the phosphorylation of PERK and eIF2α during TGF-β1 treatment (Fig. 7A, B). GSK2656157 partially prevented TGF-β1-induced morphological change of HK-2 (Fig. 7C), and dramatically reduced TGF-β1-induced expression of fibrotic marker proteins (FN, Collagen I) (Fig. 7E, G–H). GSK2656157 reduced TGF-β1-induced LC3-II expression (Fig. 7E–F), indicating the involvement of the PERK pathway in TGF-β1-induced autophagy. Meanwhile, GSK2656157 reduced apoptosis induced by TGF-β1-treatment as indicated by the reduction of TUNEL -positive cells and cleaved Caspase-3 levels (Fig. 7C–F). Together, these results support a critical role of the PERK-eIF2α pathway in TGF-β1-induced ER stress, autophagy, fibrosis, and apoptosis.

### Inhibition of autophagy enhances ER stress in HK-2 and BUMPT-306 cells during TM or TGF-β1 treatment

**Fig. 8:**
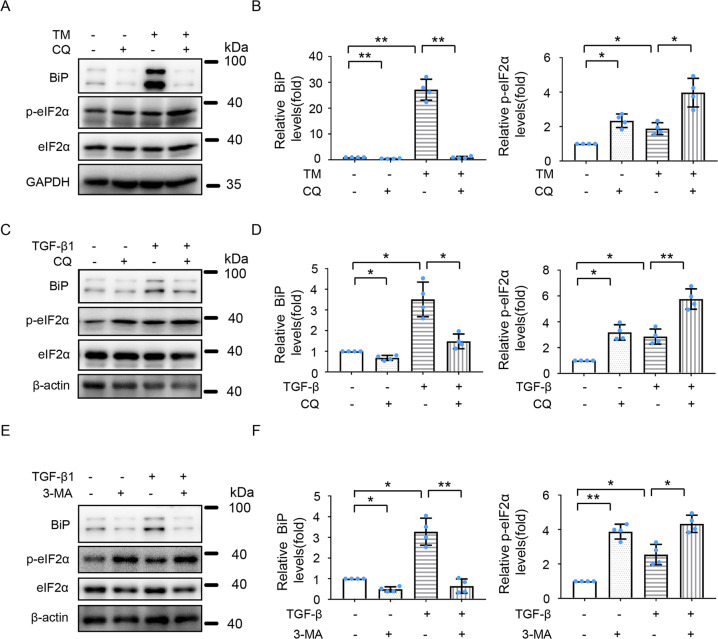
Inhibition of autophagy exaggerates ER stress during TM or TGF-β1 treatment of HK-2 cells. HK-2 cells were treated with or without 100 nM TM **A**, **B** or 5 ng/ml TGF-β1 **C**–**F** for 24 h in the absence or presence of 20 μM CQ **A**–**D** or 10 mM 3-methyladenine **E**–**F**. Cell lysates were analyzed for BiP, p-eIF2α, eIF2α and GAPDH/β-actin **A**, **C**, **E**. **B**, **D**, **F** Quantitative analysis of the intensity of BiP and p-eIF2α by densitometry. Data are expressed as mean ± SD. *n* = 4. *p < 0.05; **p < 0.01.

We further examined the effects of autophagy inhibition on ER stress in HK-2 or BUMPT-306 cells exposed to TM or TGF-β1. To this end, pharmacological inhibitors of autophagy CQ and 3-methyladenine (3-MA) were applied. As depicted in Fig. 8A–D and S4D–E, CQ enhanced TM- or TGF-β1- induced ER stress shown as BiP expression and eIF2α phosphorylation. Similar effects were demonstrated for 3-MA (Fig. 8E, F). Collectively, these results suggest that following activation, autophagy may provide a negative feedback mechanism to antagonize ER stress.

## Discussion

ER stress and autophagy have been implicated in renal fibrosis and apoptosis in kidney diseases [5, 14–18], but the relationships among ER stress, autophagy, fibrosis, and apoptosis are poorly understood. In this study, we established an ER stress-associated chronic kidney injury mouse model by repeated low dose TM treatment. Using this mouse model and renal tubular cell models injured by TM or TGF-β1, we revealed a sequential induction of ER stress, autophagy, fibrosis, and apoptosis. In cell models, blockade of ER stress by the chemical chaperone 4-PBA suppressed TGF-β1-induced autophagy, fibrosis, and apoptosis, verifying the upstream status of ER stress. In the present study, we showed that ER stress-activated autophagy, and pharmacological inhibition of autophagy dramatically reduced TM-induced renal fibrosis and apoptosis (Fig. 3 and Supplementary Fig. 4), suggesting that autophagy activation contributes at least partially to ER stress-associated renal fibrosis. Previous studies from others and us have demonstrated that autophagy activation facilitates renal fibrosis through impairing cell proliferation, inducing cell death and/or cellular senescence et al [5]. We provided further evidences that inhibition of PERK with GSK2656157 suppressed autophagy, fibrosis, and apoptosis in HK-2 and BUMPT-306 cells during TM and TGF-β1 treatment. Moreover, inhibition of autophagy by CQ and 3-MA enhanced ER stress in these cells. Together, these results indicate that ER stress may lead to the activation of autophagy through the PERK-eIF2α pathway. Upon activation, autophagy contributes to renal fibrosis and apoptosis. In addition, autophagy may antagonize ER stress, providing a negative feedback mechanism to reduce cellular stress.

The development of TIF is a complex process involving various cell types and mediators. Accumulating evidence suggests that renal proximal tubular cells play a prominent role in the development and progression of TIF. Renal tubular cells under fibrotic changes, leading to the release of pro-inflammatory and profibrotic factors that have paracrine effects on surrounding myofibroblasts and endothelia to initiate and facilitate TIF. Induction of ER stress in renal proximal tubular cells has been implicated in different experimental models of renal fibrosis, and inhibition of ER stress attenuated renal fibrosis. However, the precise role of ER stress in the initiation of TIF remains largely unclear. In the present study, we demonstrated that repeated low dose TM treatment was sufficient to induce renal fibrosis in mouse (Fig. 1D–F). Consistently, a low concentration of TM also induced fibrotic change in both human and mouse proximal tubular cells in vitro (Fig. S3 and S4C–E). However, relatively high doses of TM were lethal to mice and caused apoptosis in cultured renal tubular cells (Fig. S1 and S3A–C). These results support the concept that the severity of ER stress is critical in determining whether cells undergo fibrotic change or commit to cell death, and moreover these findings provided further evidence supporting a critical role of ER stress in the development of TIF.

The concurrence between autophagy and ER stress is common in many human pathologies, including proteinuria kidney disease, neurodegenerative disorders, cardiovascular diseases, respiratory diseases, diabetes, and cancer [8, 26]. Emerging evidence suggests an interplay between autophagy and ER stress, but the precise regulatory roles between them remain controversial. For instance, there are controversial reports on the role of ER stress in regulating autophagy. Some researchers reported that ER stress could stimulate autophagy [27–29], while others found that ER stress could inhibit autophagy [30, 31]. This discrepancy may be attributed to the varied intensity and duration of ER stress or due to the varied sensitivity of response to ER stress in different cell types. Of note, besides autophagy, ER stress and the unfolded protein response (UPR) may lead to fibrosis by inducing apoptosis, myofibroblast differentiation, epithelial-mesenchymal transition, and M2 macrophage polarization [32]. In addition, severe ER stress leads to Ca^2+^ release into the cytosol, which may result in various pathological alterations. Whether these mechanisms are also involved in ER stress-associated renal fibrosis awaits future investigation.

Hernández-Gea et al. demonstrated that ER stress induces fibrotic changes in hepatic stellate cells through activating autophagy [33]. In that study, activation of the IRE1-Xbp1 pathway was shown to trigger autophagy in a p38-dependent manner. Currently, it keeps unclear through which signaling pathway ER stress activates autophagy in kidneys during renal fibrosis. Recent studies suggest a critical role of the PERK-eIF2α pathway in mediating the interlinking of ER stress or UPR to autophagy [34–36]. PERK is a key determinant of cell fate upon ER stress. Short-term induction of PERK seems to promote cell survival by blocking protein translation via inducing eIF2α phosphorylation, whereas prolonged activation of PERK triggers apoptosis [37]. The PERK-eIF2α pathway has been reported to participate in fibrosis in other organs such as the liver, lung, and heart [38–40]. In our study, inhibition of PERK reduced TM- or TGF-β1- induced autophagy in HK-2 and BUMPT-306 cells, leading to notable decreases in fibrogenic response and apoptosis. These results support a critical role of the PERK-eIF2α pathway in autophagy activation, fibrotic changes, and apoptosis during ER stress in renal tubular cells. Accordingly, targeting this pathway may have a therapeutic potential to reduce renal fibrosis and apoptosis in kidney diseases. Also, ER stress may release Ca^2+^ from ER to activate CaMKKβ/AMPK, inhibit mTORC1, or activate death-associated protein kinase 1 for autophagy [41]. Whether these mechanisms contribute to autophagy activation by ER stress in renal fibrosis remains to be investigated.

Regarding the regulatory role of autophagy on ER stress, only a few studies have been reported. Generally, autophagy impairment or inhibition could activate ER stress because of the accumulation of misfolded/unfolded proteins and protein aggregates due to the lack of autophagic degradation [42, 43]. In sharp contrast, Li et al. showed that inhibition of autophagy may attenuate ER stress [44]. In our study, inhibition of autophagy with CQ and 3-MA further enhanced TM- or TGF-β1-induced ER stress in HK-2 and BUMPT-306 cells, indicating that upon activation, autophagy can antagonize or relieve ER stress. This observation is consistent with the notion that autophagy may reduce ER stress by degrading protein aggregates. In addition, autophagy may selectively remove or degrade damaged or dysfunctional ER via ER-Phagy.

In summary, this study provides both in vitro and in vivo evidence that ER stress in renal proximal tubular cells is sufficient to induce renal fibrosis. The PERK-eIF2α UPR pathway is involved in linking ER stress to the autophagy activation, and autophagy activation at least partially contributes to ER stress-induced renal fibrosis. Moreover, inhibition of autophagy exaggerated ER stress, suggesting that autophagy induced in ER stress provides a negative feedback mechanism to reduce the stress. Collectively, the present study unveils a reciprocal regulation between ER stress and autophagy in chronic kidney injury and fibrosis. Despite these interesting findings, the precise mechanism mediating the interplay between ER stress and autophagy, and their contribution to renal fibrosis awaits deeper investigation.

## Materials and methods

### Mouse model of TM-induced fibrosis

C57BL/6 mice (male, 8–10 weeks) were purchased from Hunan SJA Laboratory Animal Corporation (Changsha, China). These mice were housed in a pathogen-free animal facility at the Second Xiangya Hospital in a 12 h light-dark cycle with free access to water and food. The mice were randomly divided into groups and intraperitoneally injected with one dose of 1, 2, and 4 mg/kg Tunicamycin (TM) (ab120296, Abcam, Cambridge, UK), or with two weekly doses of 0.25 or 0.5 mg/kg TM. For control mice, the vehicle solution of 5% dimethyl sulfoxide (DMSO) was injected. The mice were sacrificed at day 7, 14, or 21 after drug injection.

### Cell culture

The immortalized human proximal tubular epithelial HK-2 cell line was cultured in DMEM/F-12 supplemented with 10% fetal bovine serum (FBS), 1% L-glutamine, 1% sodium pyruvate, and 1% penicillin-streptomycin solution. The mouse renal proximal tubular epithelial cell line BUMPT-306 cells were originally obtained from Dr. Wilfred Lieberthal (Boston University School of Medicine) and cultured as described previously [16]. For TM treatment, cells were starved overnight in serum-free DMEM/F-12 medium at ~40–50% confluence, and then incubated with the indicated concentrations of TM and harvested at indicated time-points. Control cells were kept in a serum-free medium containing DMSO. For TGF-β1 treatment, cells were starved overnight at 30−40% confluence and then treated with the indicated concentrations of TGF-β1 and harvested at different time points. For pre-treatment, 20 μM chloroquine (CQ,) (C6628, Sigma-Aldrich, St. Louis, Missouri, USA), 1 μM GSK2656157 (5.04651, EMD Millipore, Massachusetts, USA), 1 mM 4-phenyl butyric acid (4-PBA) (S4125, Selleck, Houston, TX, USA), or 10 mM 3-methyladenine(3-MA) (M9281, Sigma-Aldrich, St. Louis, Missouri, USA) was added 1 h before adding100 nM TM or 5 ng/ml TGF-β1 (GF111, EMD Millipore, Massachusetts, USA), and the cells were treated for 24 h.

### Analysis of autophagy by transfecting mRFP-GFP-LC3

HK-2 cells were transiently transfected with mRFP-GFP-LC3 (ptfLC3, Addgene plasmid 21074) using Lipofectamine 3000 (Invitrogen, California, USA) according to a standard procedure. The cells were treated at 24 h after the transfection. RFP and GFP fluorescence images were collected by fluorescence microscopy. For quantification, approximately 100 fluorescently labeled cells from 10 to 20 random fields were counted in each condition.

### Immunoblot analysis

Renal tissues or HK-2 cells or BUMPT-306 cells were lysed using 2% sodium dodecyl sulfate (SDS) buffer containing 1% protease inhibitor cocktail (Sigma-Aldrich, St. Louis, Missouri, USA). Protein concentration was estimated using a Pierce BCA Protein Assay Kit. Proteins were loaded and separated by SDS- polyacrylamide gel, and then transferred to polyvinylidene difluoride (PVDF) membranes. The PVDF membranes were blocked in 5% bovine serum albumin or 5% fat-free milk and subsequently incubated with primary antibodies at 4 °C overnight and secondary antibodies. Band density for the protein of interest was normalized to either GAPDH or β-actin. The following antibodies were used in this study: anti-BiP(3177 S), anti-p-PERK(3179 S), anti-PERK(3192 S), anti-p-eIF2α (3597 S), anti-eIF2α (5324 S), anti-p62 (39749 S), anti-Cleaved-Caspase-3 (9664 S) from Cell Signaling Technology (Boston, USA); anti-LC3 (NB100-2220) from Novus Biologicals (Colorado, USA); anti-Fibronectin (FN) (ab2413) from Abcam (Cambridge, UK); anti-Collage I (AF7001) from Affinity (Jiangsu, China); anti-β-actin (60008-1-Ig) and anti-GAPDH (10494-1-AP) from Proteintech (Chicago, USA). Secondary antibodies were purchased from Thermo Fisher Scientific (Waltham, Massachusetts, USA).

### Immunohistochemistry staining

Paraffin-embedded kidney tissue sections of 4 μm thickness were deparaffinized and endogenous peroxidase was inactivated with H_2_O_2_. Sections were then blocked in 2% goat serum for 1 h at room temperature and then incubated with anti-FN antibody (ab2413, Abcam, Cambridge, UK) at 4 °C overnight. The next day, sections were washed 3 times with PBS and then incubated with secondary antibody for 30 m. The antigen-antibody complexes signals were developed with a DAB kit. Finally, the kidney tissues were counterstained with hematoxylin.

### Immunofluorescence staining

Paraffin-embedded kidney tissue sections of 4 μm thickness were deparaffinized and incubated with EDTA for antigen retrieval. The following steps were performed using Tyramide SuperBoost™ Kits with Alexa Fluor™ Tyramides (Thermo Fisher Scientific, Waltham, Massachusetts, USA). The slides were incubated with 3% Hydrogen Peroxide Solution at room temperature for 1 h. After washing with PBS, kidney tissues were blocked for 1 h with blocking buffer. Then the tissues were exposed to 1:5000 anti-LC3 (NB100-2220, Novus Biologicals, Colorado, USA) and 1:50 anti-Megalin (sc-515772, SANTA CRUZ BIOTECHNOLOGY, California, USA) at 4 °C overnight and incubated with poly-HRP-conjugated secondary antibody and goat anti-mouse IgG conjugated with Alexa Fluor 594 for 1 h at room temperature. After washing with PBS, the slides were incubated with a tyramide working solution and Reaction Stop Reagent for 2.5 m each at room temperature. For quantification, 10 to 20 random fields (X630 magnification) were selected for each tissue and the dots of LC3B per tubule were evaluated; HK-2 cells were washed with phosphate-buffered saline (PBS) and fixed with cold methanol: acetone (1:1) at room temperature for 10 m. After washing with PBS, fixed cells were blocked for 30 m with 10% normal goat serum. Then the cells were incubated with anti-Collagen I (AF7001, Affinity, Jiangsu, China)) overnight at 4 °C and exposed to goat anti-rabbit IgG conjugated with Alexa Fluor 594 for 1 h at room temperature. Hoechst 33342 was used as a nuclear counterstain.

### Masson trichrome staining

Masson trichrome staining was performed on paraffin-embedded kidney tissue to evaluate collagen fibrils with the reagents from Servicebio according to a standard procedure. Collagen I, collagen III, and collagen IV were stained with aniline blue.

### Tdt-mediated dUTP nick-end labeling (TUNEL) assay

The In Situ Cell Death Detection Kits (Roche Life Science, Switzerland) were used to detect the apoptotic cells on 8-μm-thick frozen kidney sections or HK-2 cells following the manufacturer’s instructions. Briefly, frozen tissue sections and fixed HK-2 cells were pretreated with 0.1 M sodium citrate for 30 min at room temperature and incubated in a dark, humidified chamber for 1 h with TUNEL reaction mixtures at 37 °C. For kidney tissue, 10 optical fields from each frozen tissue section were obtained and quantification of TUNEL- positive cells per mm^2^ was performed. For HK-2 cells, 10 optical fields from four different experiments were examined to evaluate the apoptosis percentage.

### Quantification of immunostaining

Image J software was used to measure the level of immunostaining in the renal cortex. First, the images were converted to 8-bit grayscale. Positive staining regions were selected for measurement of area and integrated density, and the background intensity was measured by selecting 3 distinct areas in the background with no staining.

### Statistics

All in vivo qualitative data are representatives of at least 6 individual animals and in vitro qualitative data are representatives of at least 4 independent experiments. Morphological analysis was done in a blinded manner. The data were expressed as means ± SD. GraphPad Prism 7 software was used for statistical analyses. A two-tailed unpaired Student *t*-test was used to analyze differences between two groups. One-way or two-way ANOVA was used to analyze differences in more than 2 groups. *P* < 0.05 was regarded as significantly different.

## Supplementary information

Supplementary Legends

Supplementary Figure 1

Supplementary Figure 2

Supplementary Figure 3

Supplementary Figure 4

Supplementary Figure 5

Supplementary Figure 6

Supplementary Figure 7

## Data Availability

All data generated or analysed during this study are included in this published article [and its supplementary information files].
