# Supplementary material for: Reciprocal regulation between ER stress and autophagy in renal tubular fibrosis and apoptosis
Source: Cell Death Dis. 2021 Oct 29;12(11):1016. doi: 10.1038/s41419-021-04274-7 (PMC8556380; doi:10.1038/s41419-021-04274-7)
Supplement: Supplementary file 1 — Supplementary Legends [file 41419_2021_4274_MOESM1_ESM.docx]

**Supplementary Figure Legends**

**Supplementary Fig. 1**. **(A) Survival rate of the mice receiving relatively high doses of TM.** C57BL/6 mice (male, 8–10 weeks) were injected with one dose of 1, 2, and 4 mg/kg of TM or DMSO intra-peritoneally. The mice were monitored for 21 days after the injection.

**Supplementary Fig. 2. TM induces autophagy in renal proximal tubular cells in mice.** C57BL/6 mice (male, 8-10 weeks) were subjected to two weekly injections of 0.25 mg/kg TM (or DMSO for control) to collect kidney tissues 7 days later. (**A**) Representative immunofluorescence images of LC3 (green) and Megalin (to label proximal tubular cell) (red). Bar=20 μm.

**Supplementary Fig. 3.** **Induction of ER stress, autophagy, fibrosis and apoptosis by TM in HK-2 cells.** HK-2 cells were exposed to different concentrations of TM (0, 50, 100, 200 and 300 nM) for 24 h. DMSO was used as vehicle control. (**A**) Representative phase contrast images showing HK-2 cells morphology. Scale bar=100 μm. (**B**) Cell lysates were analyzed for BiP, LC3, p62, FN, Collagen I, cleaved Caspase-3, and GAPDH by immunoblot analysis. (**C**) Densitometric analysis of immunoblots. (**D**) Representative images of Collagen I immunofluorescence. Scale Bar=100 μm. (**E**) Quantitative analysis of Collagen I immunofluorescence intensity. Data are expressed as mean ± SD. n = 4. *p < 0.05; **p < 0.01; ***p < 0.001; ns, not significant.

**Supplementary Fig. 4. Inhibition of autophagy attenuates TM -induced fibrosis and apoptosis and exaggerates ER stress in BUMPT-306 cells.** BUMPT-306 cells were treated with or without TM (100 nM) in the absence or presence of CQ (20 μM) for 24 h. (**A, B**) Cell lysates were analyzed for LC3 by immunoblot analysis and quantified by densitometry. (**C**) Representative phase contrast images of BUMPT-306 cells. Scale bar=50 μm. (**D, E**) Cell lysates were analyzed for levels of FN, Cleaved Caspase-3, p-eIF2α, eIF2α and GAPDH by immunoblot analysis and quantified by densitometry. Quantitative data are expressed as mean ± SD. n = 4. *p < 0.05; ***p < 0.001; ****p < 0.0001.

**Supplementary Fig. 5. Inhibition of PERK mitigates autophagy, fibrosis and apoptosis during TM treatment of** **BUMPT-306 cells.** BUMPT-306 cells were treated with or without TM (100 nM) in the absence or presence of 1 μM GSK2656157 for 24 h. (**A**) Cell lysates were analyzed for p-PERK, PERK, p-eIF2α, eIF2α and GAPDH by Immunoblot analysis; (**B**) Densitometric analysis of p-PERK and p-eIF2α band signals. (**C**) Representative phase contrast images of BUMPT-306 cells. Scale bar=50 μm. (**D**) Cell lysates were analyzed for LC3-II, FN, cleaved Caspase-3, and GAPDH by Immunoblot. (**E**) Densitometric analysis of LC3-II, FN and cleaved Caspase-3 intensity. Data are expressed as mean ± SD. n = 4. ***p < 0.001; ****p < 0.0001.

**Supplementary Fig. 6. Induction of ER stress, autophagy, fibrosis and apoptosis in HK-2 cells by TGF-β1.** HK-2 cells were subjected to different concentrations of TGF-β1 (0, 1, 2, 5 and 10 ng/ml) for 24 h. (**A**) Representative phase contrast images showing cell morphology. Scale Bar=100 μm. (**B**) Cell lysates were analyzed for BiP, LC3, FN, Cleaved Caspase-3, and β**-**actin by immunoblot analysis. (**C**) Densitometry analysis of proteins relative to β-actin; (**D**) Representative images of Collagen I. Scale bar=100 μm. (**E**) Quantitative analysis of Collagen I immunofluorescence intensity. Data are expressed as mean ± SD. n = 4. *p < 0.05; **p < 0.01; ns, not significant.

**Supplementary Fig. 7. Sequential induction of ER stress, autophagy, fibrosis and apoptosis in HK-2 cells by TGF-β1.** (**A, B**) HK-2 cells were subjected to 5 ng/ml of TGF-β1 for indicated time (0, 1, 2, 4, 8 and 24 h). Cell lysates were analyzed for BiP, LC3, FN and Cleaved Caspase-3 by immunoblotting and quantified by densitometry. (**C-E**) HK-2 cells were transfected with RFP-GFP-LC3 and then treated with 5 ng/ml of TGF-β1 for 2 h. (**C**) Representative fluorescence images showing GFP and RFP LC3 puncta in HK-2 cells. Scale bar=10 μm; (**D**) Quantitative analysis of GFP and RFP LC3 puncta. Data are expressed as mean ± SD. ^#^P<0.05 vs control, ^P<0.05 vs GFP-LC3 puncta in TGF-β1 group. (**E**) Analysis of autophagic flux rate. (**F**) Representative images of Collagen I immunofluorescence and TUNEL staining. Scale Bar=100 μm. (**G**) Quantitative analysis of Collagen I immunofluorescence signal. (**H**) Quantitative analysis of TUNEL positive cells. Data are expressed as mean ± SD. n = 4. *p<0.05; **p<0.01; ns, not significant.
