# Supplementary figures and images for: Reciprocal regulation between ER stress and autophagy in renal tubular fibrosis and apoptosis
Source: Cell Death Dis. 2021 Oct 29;12(11):1016. doi: 10.1038/s41419-021-04274-7 (PMC8556380; doi:10.1038/s41419-021-04274-7)

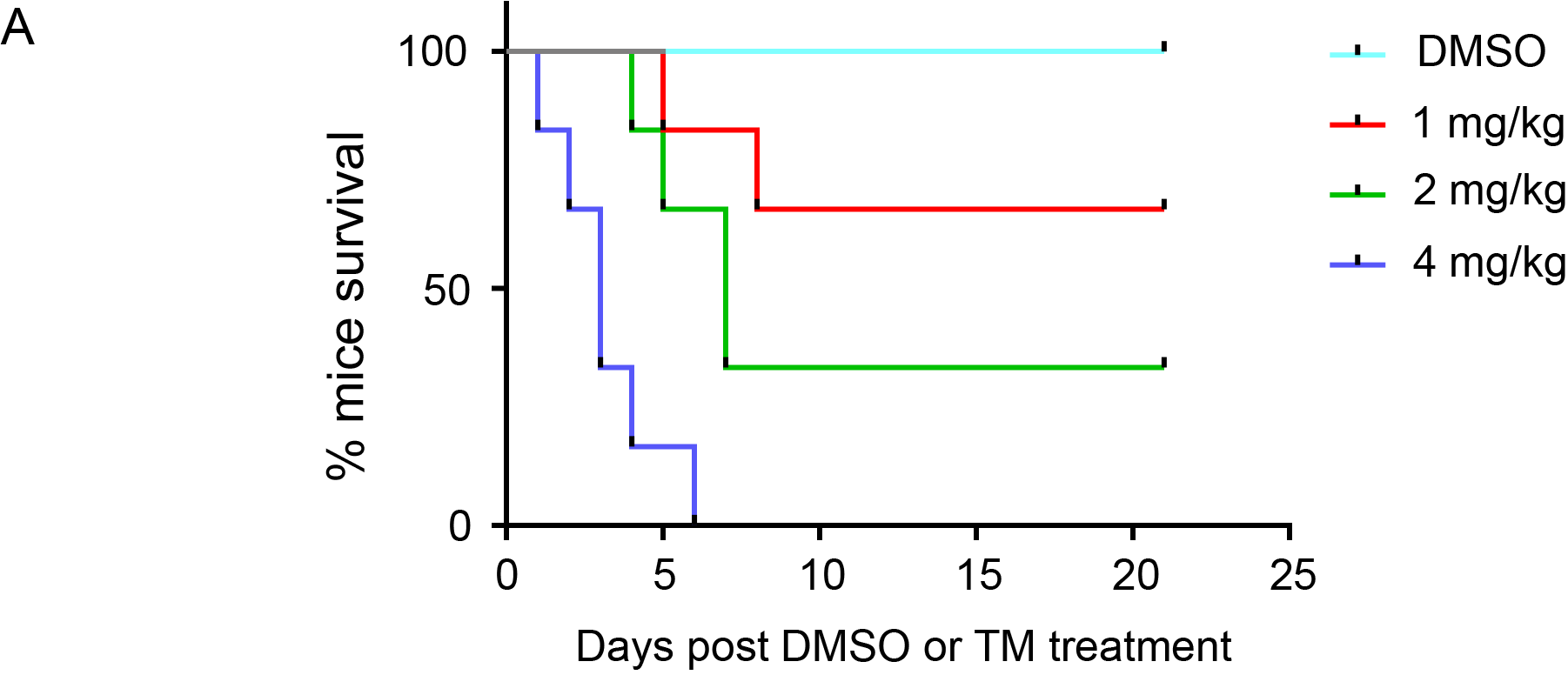

Supplement: Supplementary file 2 — Supplementary Figure 1 [file 41419_2021_4274_MOESM2_ESM.png]

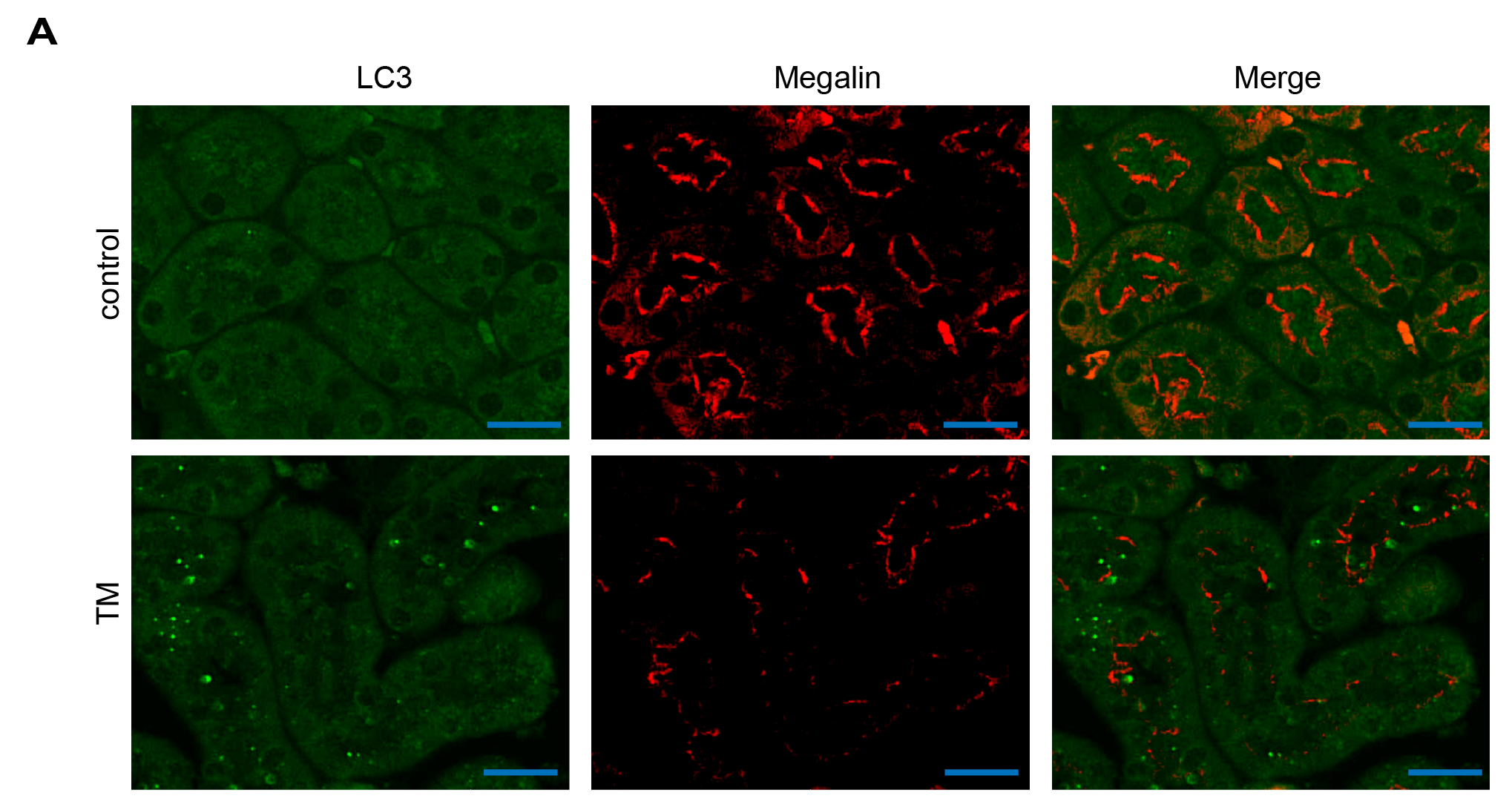

Supplement: Supplementary file 3 — Supplementary Figure 2 [file 41419_2021_4274_MOESM3_ESM.png]

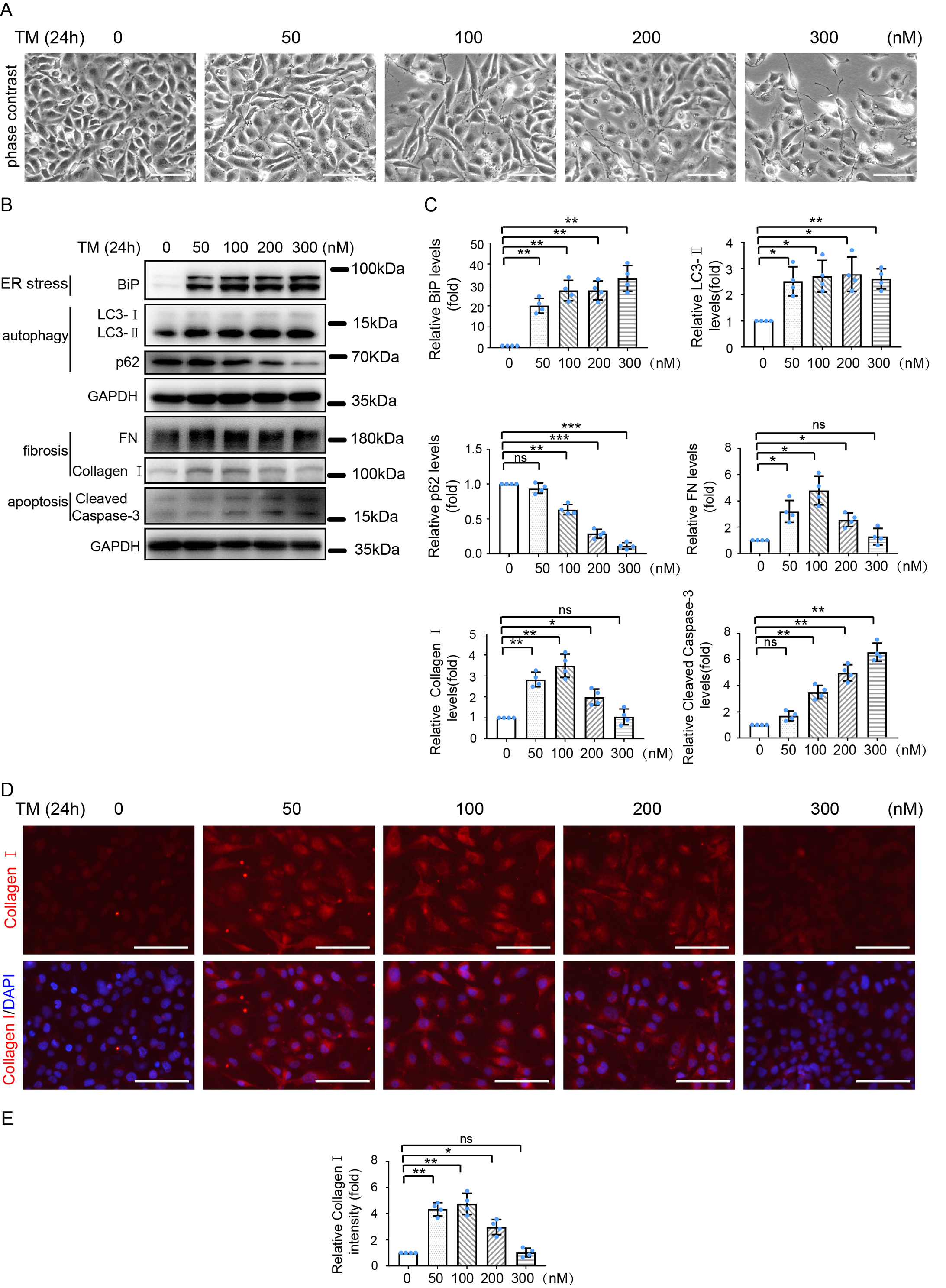

Supplement: Supplementary file 4 — Supplementary Figure 3 [file 41419_2021_4274_MOESM4_ESM.png]

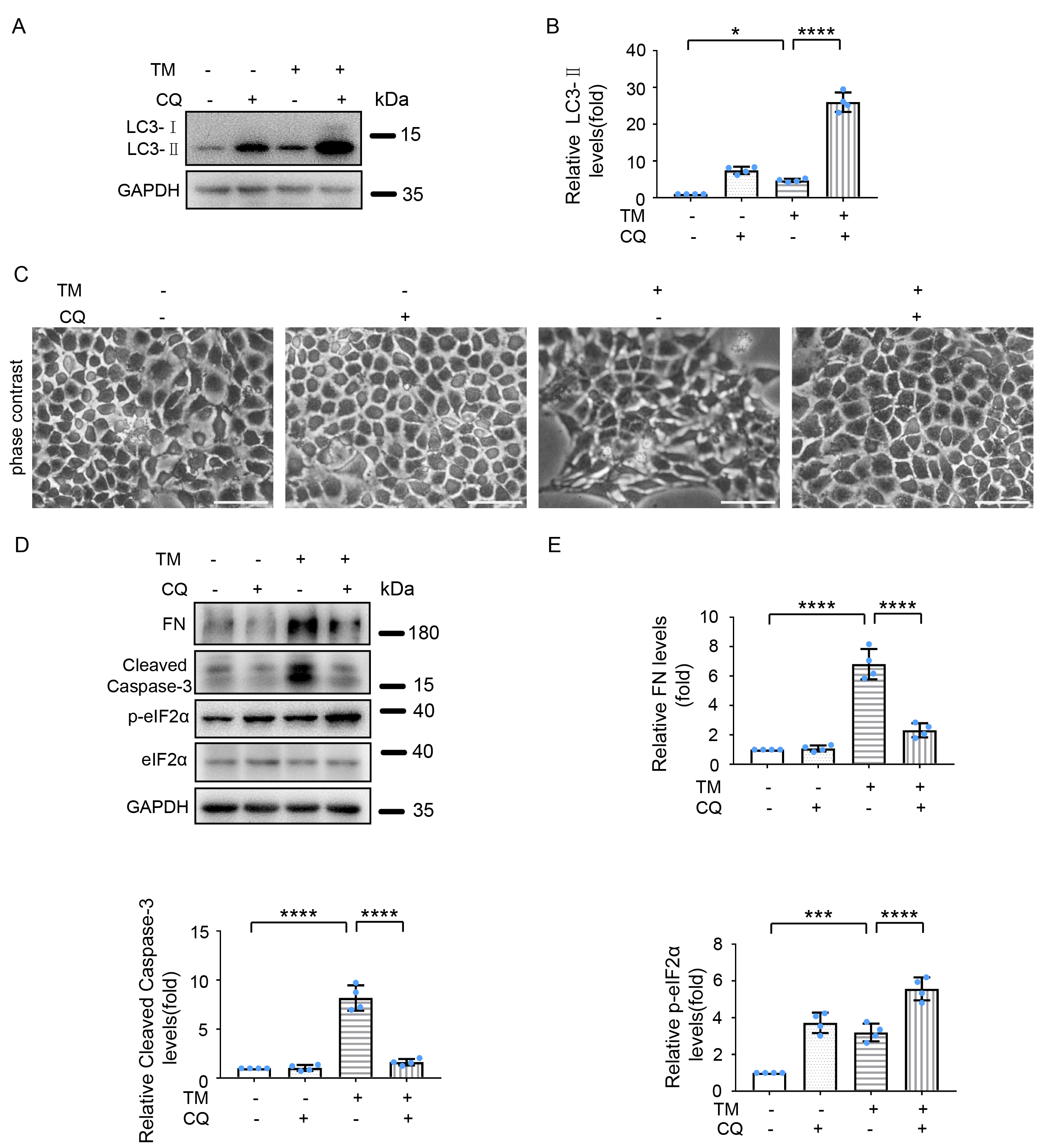

Supplement: Supplementary file 5 — Supplementary Figure 4 [file 41419_2021_4274_MOESM5_ESM.png]

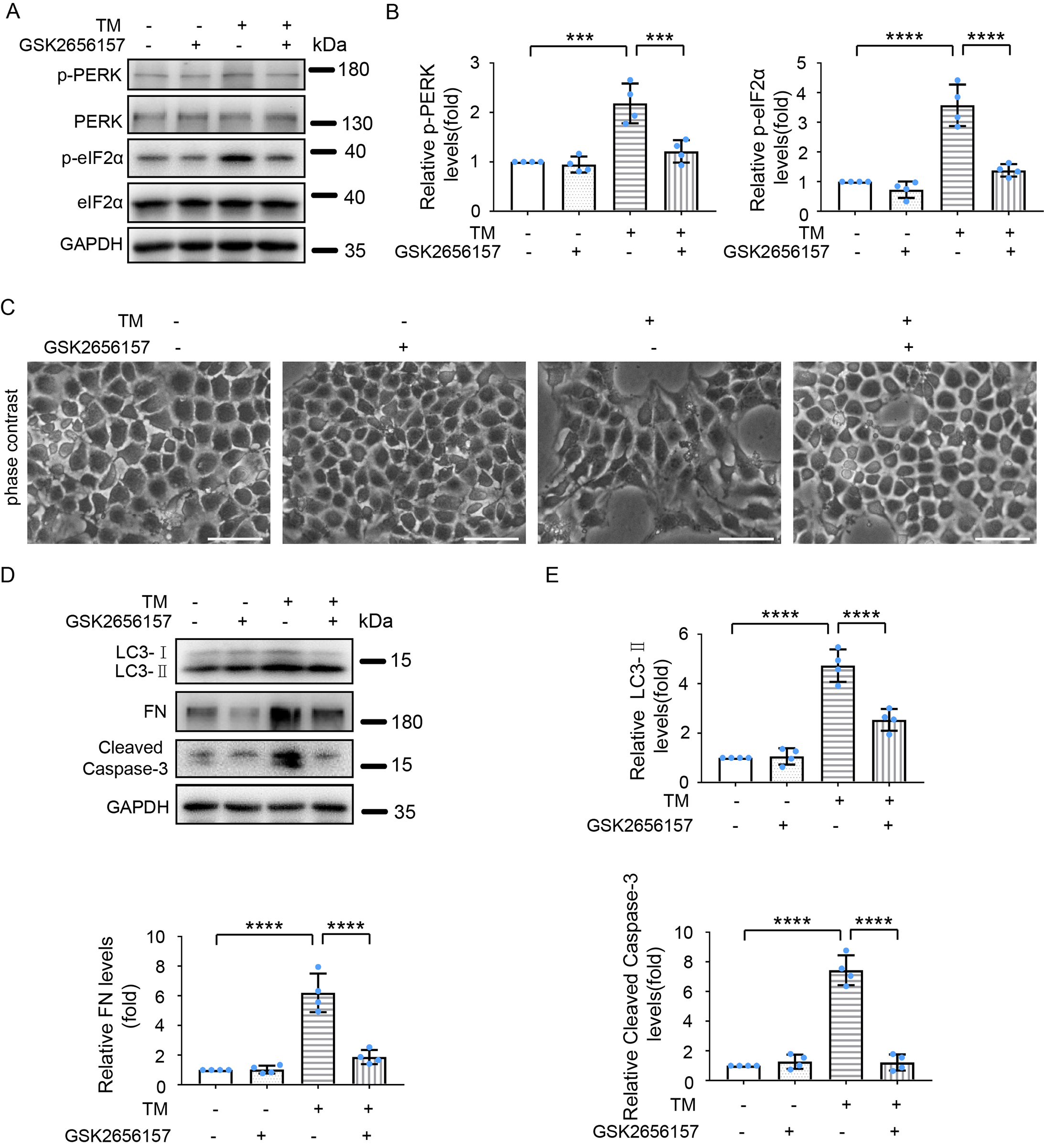

Supplement: Supplementary file 6 — Supplementary Figure 5 [file 41419_2021_4274_MOESM6_ESM.png]

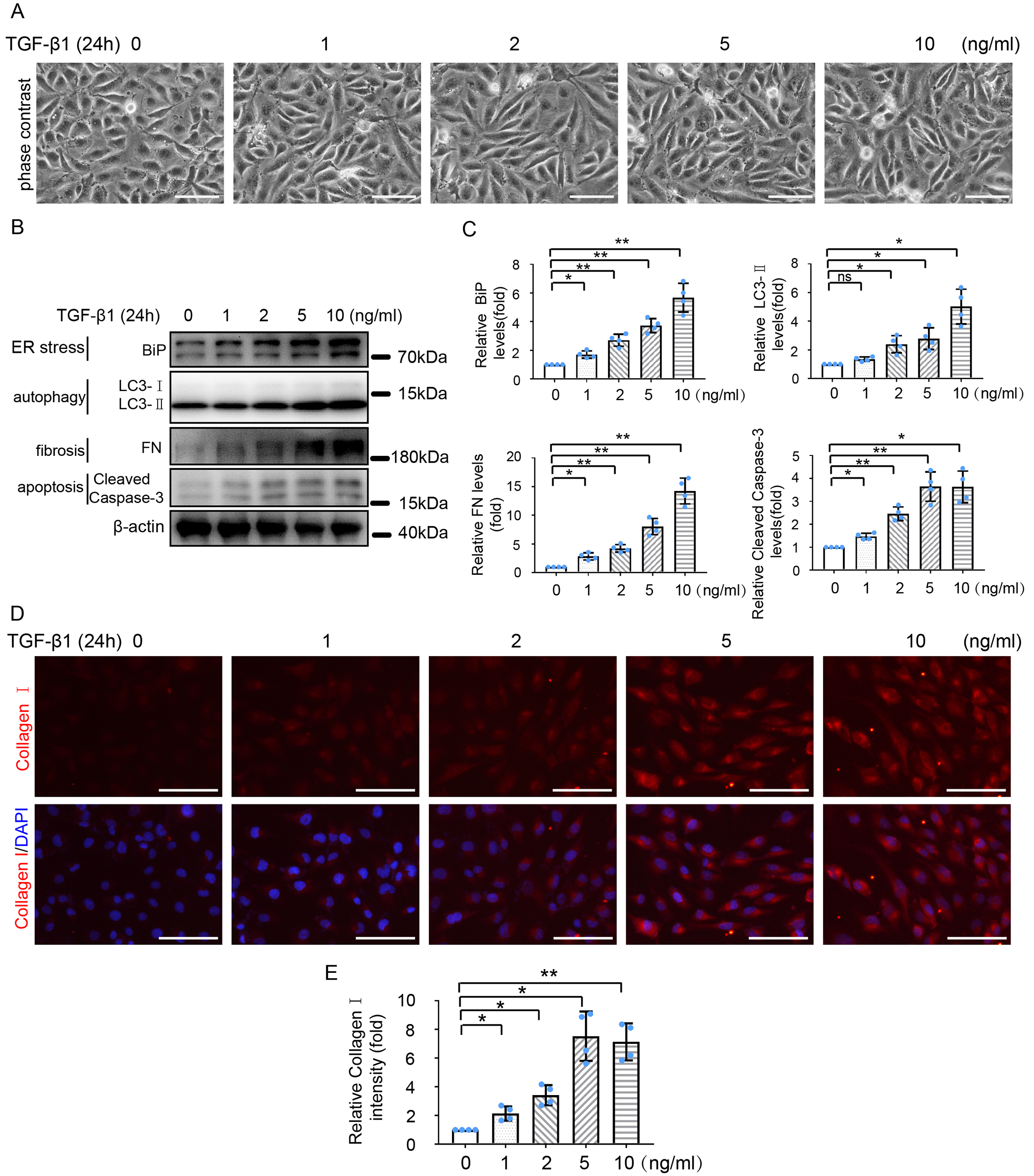

Supplement: Supplementary file 7 — Supplementary Figure 6 [file 41419_2021_4274_MOESM7_ESM.png]

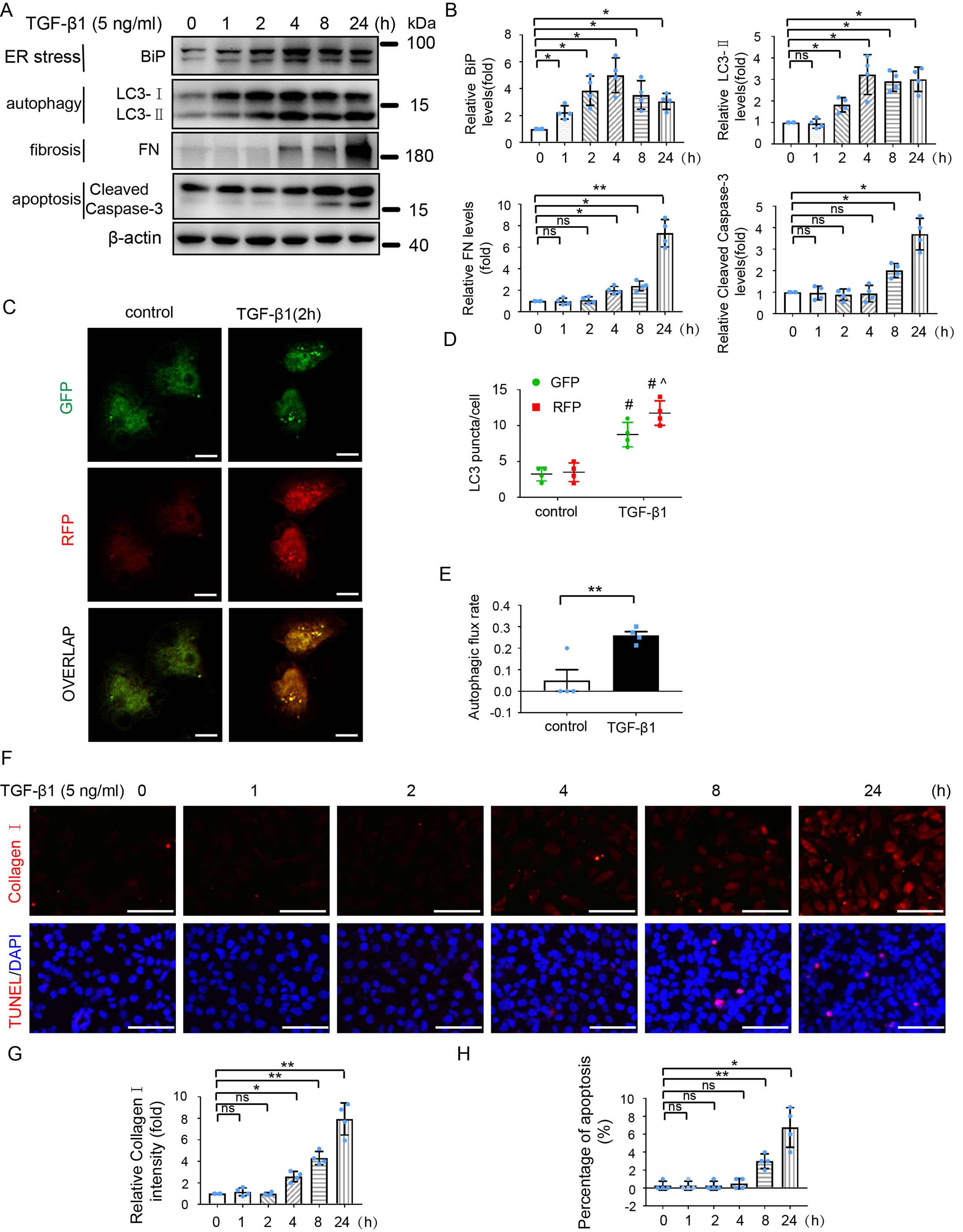

Supplement: Supplementary file 8 — Supplementary Figure 7 [file 41419_2021_4274_MOESM8_ESM.png]
